# Combined Experimental and Machine Learning Study on the Interplay between Delignification and Mechanical Properties for Improved Poplar Wood Reconstruction

**DOI:** 10.1021/acsami.5c20194

**Published:** 2026-01-14

**Authors:** A. Vahid Movahedi-Rad, Maximilian Ritter, Alan Colmant, Dan Vivas Glaser, Sandro Stucki, Ingo Burgert, Guido Panzarasa

**Affiliations:** † Wood Materials Science, Institute for Building Materials, 27219 ETH Zürich, Zürich 8093, Switzerland; ‡ Laboratory for Cellulose and Wood Materials, Group WoodTec, Empa, Swiss Federal Laboratories for Materials Science and Technology, Überlandstr. 129, Dübendorf 8600, Switzerland

**Keywords:** biocomposite, delignification, mechanical properties, water uptake, life cycle assessment (LCA), machine learning

## Abstract

Structure-retaining delignification of wood is widely used to obtain scaffolds suitable for the preparation of high-performance biobased composites. However, this often comes at the expense of sustainability and large-scale production potential. To address these issues, we reconstructed poplar wood via room-temperature partial delignification, followed by delignification and densification. Compared to fully delignified samples, those obtained with partial delignification have superior mechanical properties at 45° and 90° fiber directions with respect to the loading direction, but lower ones at 0°. Working at room temperature facilitated sample up-scaling and allowed reuse of the delignification solution multiple times without compromising product quality. As shown by life cycle assessment (LCA), the possibility of repeatedly reusing the delignification solution led to a significant reduction in the global warming potential (GWP) and ecosystem quality (EQ) impacts. We then developed an ’unsupervised, supervised classification, supervised regression’ (USS) learning framework to accurately predict the mechanical properties of reconstructed poplar on the basis of structural and process-related features, followed by feature importance analysis to determine the key parameters influencing material performance. With our approach, we were able to estimate the mechanical performance of the reconstructed samples and gain insight into the most relevant material-fabrication parameters.

## Graphical abstract

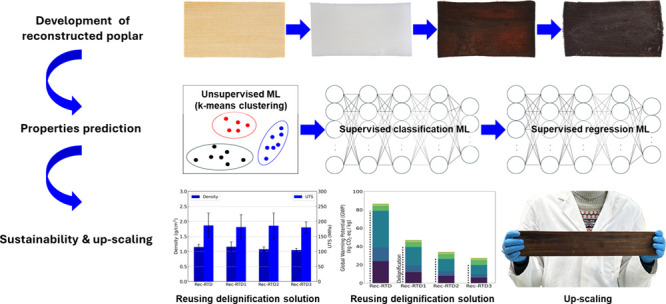

## 1 Introduction

Concerns over the harmful environmental impacts of petroleum-based polymers, from their production to their degradation byproducts, e.g., microplastics, as well as the uncertainty surrounding future fossil fuel supplies, are growing.^1,2^ As a result, there is an increasing interest in developing high-performance eco-friendly composite materials from renewable resources for a variety of commercial uses.^3,4^ Wood is a widely available natural resource that has been used for thousands of years as a structural component for buildings and furniture.^5–7^ However, compared to several artificial high-performance fiber-reinforced engineering materials, the applicability of wood is limited by its tendency to undergo moisture-induced swelling and shrinkage, as well as its lower mechanical performance, especially in the wet state.^8^ These challenges are further compounded by the variability in wood’s internal structure and density, along with as-growth imperfections, e.g., knots, which directly influence its mechanical properties and limit its widespread use for high-performance applications.^9^

An effective strategy to address these concerns is through structure-retaining delignification, which facilitates the impregnation of resins into the wood scaffold and enhances the efficiency of densification.^10–14^ By using this method, the fiber orientation of the natural wood is preserved, leading to an improvement in the mechanical properties.^15^ Most delignification protocols are based on acidic chlorite^16^ or alkaline sulfite.^17^ Frey et al.^10^ proposed a protocol to achieve structure-retaining, almost full delignification of bulk wood using a mixture of concentrated hydrogen peroxide and acetic acid at a high temperature of 80 °C. The resulting delignified wood could be efficiently impregnated with resins and densified, resulting in composites with enhanced mechanical properties. The great potential of fully delignified wood motivated other researchers to develop new biobased composites with more homogeneous properties.^11,14,15,18^ However, full delignification could require the use of concentrated acids and peroxides at high temperatures, an approach that presents significant challenges for industrial scale-up. Moreover, the resulting cellulose scaffold is relatively fragile, making the handling of wet delignified wood challenging.^9^ However, partial delignification of wood, combined with resin impregnation and hot pressing at proper moisture content, can also result in strong natural fiber-reinforced composites.^12,13,17,19^ Alqrinawi et. al^13^ developed a new composite with significantly improved mechanical properties by partially delignifying low-density balsa wood followed by a chemical impregnation treatment and hot pressing.

The use of petroleum-based resins to impregnate delignified wood^11,15,19–22^ is a widely used approach, which, however, negatively affects the sustainability and biodegradability of the final product.^12^ Lignin and lignin-based resins show potential for partially or completely replacing petrochemical-based resins.^23–29^ Lignin is a byproduct of pulp and paper production, abundant, environmentally friendly, and biodegradable, making it an ideal candidate for developing more sustainable biobased composites.^14,27^

Despite significant progress in developing biobased wood composites, several critical gaps remain. First, the large-scale fabrication of structure-retaining wood-based composites is hindered by impractical functionalization protocols and low processing efficiency, which limit their wider practical implementation.^30^ Second, for most practical applications, fiber-reinforced composites are often subjected to multidirectional loads, where both the matrix and the fiber–matrix interface play a significant role in determining the load-bearing capacity and resistance to deformation.^31,32^ For this reason, characterizing the mechanical properties of these composites only in the fiber direction, where fibers effectively transfer and bear the applied stress, does not allow for capturing the anisotropic behavior necessary to meet performance criteria for the intended applications.^31,33–35^ Jakob et al.^36^ reported that, although partially delignified and densified spruce-based composites exhibited improved tensile strength and modulus of elasticity along the fiber direction (0°), their transverse (90°) performance was inferior to that of native wood. This limitation must be considered when evaluating the suitability of such materials for load-bearing applications. Literature studies primarily focus on mechanical properties along the 0° fiber direction, while the off-axis behavior, particularly its low ductility, remains insufficiently addressed. There is also a need for biobased materials that combine high water stability and fire resistance to ensure reliability under real-world conditions. Furthermore, although many existing approaches involve the use of biobased materials, their fabrication processes are not always sustainable, highlighting the importance of developing methods that enable the reuse of materials and chemicals, with impacts verified quantitatively through life cycle assessment (LCA). Last but not least, integrating predictive modeling to estimate material properties based on fabrication parameters could significantly accelerate material development while minimizing experimental efforts.

Machine learning algorithms can be used for extracting meaningful patterns from complex and diverse data sets, enabling the development of efficient predictive models.^37–40^ These models reduce the reliance on extensive experimental testing, thereby accelerating the evaluation and optimization of different types of research, including in wood-based materials.^41,42^ Unlike traditional material design, which is constrained by low-dimensional design spaces, machine learning methods enable exploration of high-dimensional, multivariable relationships such as those involved in material development, nondestructive testing, prediction of material properties, and of processing conditions.^43^ A notable example in the framework of biocomposite development is represented by the report of Yang and Qin,^38^ who integrated their experimental findings with a machine learning model to develop lightweight and high-strength mycelium-based wood composites, enabling multiphysics predictions within a high-dimensional design space that could not be fully explored by experimental methods alone.

In this work, we aim to develop a high-performance biobased fiber-reinforced composite using a protocol that could allow its scalable production in an environmentally sustainable way, which was evaluated through life cycle assessment (LCA). As in our previous study on wood reconstruction,^14^ we use poplar wood (a low-grade wood species underutilized for structural applications) but apply a partial delignification protocol to address the challenges associated with extensive delignification, especially toward up-scaling. In addition, we implement an ‘unsupervised, supervised classification, supervised regression (USS)’ learning framework to predict the elastic modulus and strength of reconstructed wood composites. Our USS learning framework consists of (i) a k-means clustering algorithm, (ii) a classifier supervised machine learning, and (iii) a regressor supervised machine learning. Feature importance analysis was also conducted to identify the most influential structural and processing parameters. The properties of the obtained reconstructed poplar were evaluated based on mechanical properties under various loading directions, water resistance, and fire resistance. This work outlines a procedure for developing a biobased composite addressing challenges of practical relevance.

## 2 Materials and Methods

### 2.1 Materials

Poplar (Populus spp.) wood samples (30 × 60 mm^2^, 4-mm-thick) were cut along the longitudinal direction, with a growth ring inclination of around 45°. Hydrogen peroxide solution (30%) and citric acid (anhydrous) were purchased from Fisher Chemicals. Sodium hydroxide, acetic acid (glacial), and isopropanol were purchased from Sigma-Aldrich. Lineo Classic W, a lignin separated during the kraft pulping process of Nordic softwood, was generously provided by Stora Enso (Finland) (raw materials: spruce *Picea abies* and pine *Pinus sylvestris*, lignin dry content 64 ± 6%, ash <2.5%, sulfur >3.0%, residual carbohydrates <2.0%, pH of a 40% slurry in water 2–4, bulk density 550–650 kg m^–3^, average molecular weight Mw 5500–7500 Da according to the producer).

### 2.2 Preparation of Water-Soluble Lignin

In a typical preparation, 30 g of powdered Kraft lignin was added in small portions under stirring (500 rpm) to 60 mL of a NaOH 2.5 M aqueous solution. As the lignin dissolved, a dark-brown solution was formed. When all of the lignin was dissolved, the solution was slowly added under continuous stirring to 600–700 mL of isopropanol. The lignin sodium salt precipitated immediately as a black solid, which could be decanted from the orange supernatant and collected by vacuum filtration. The powder was then dried at 40 °C under a vacuum until a dark-brown, free-flowing powder was obtained. The resulting water-soluble lignin was stored in a closed container until use.

### 2.3 Preparation of Reconstructed Poplar

A delignification solution was prepared as a 50:50 v/v mixture of concentrated hydrogen peroxide and glacial acetic acid. Room-temperature delignification (RTD) was carried out by submerging native wood in the delignification solution at room temperature for 16 h. To achieve higher levels of delignification, room-temperature delignification was followed by a hot delignification step at 80 °C for 2 h (2HD) or for 6 h (6HD). Afterward, the specimens were rinsed several times with deionized water until they reached pH 6.0. The specimens were stored under ambient conditions for 24 h before being impregnated with water-soluble lignin. Water-soluble lignin (prepared as described in Section 2.2) was dissolved in deionized water with a weight ratio of 1:5. The delignified wood specimens were impregnated with a lignin solution at room temperature for 24 h. Afterward, the samples were kept under the fume hood for 2 days, and then, they were submerged into a solution made by dissolving citric acid in deionized water with a weight ratio of 1:5 for 1 h at room temperature to precipitate lignin into the delignified wood scaffold (via a “quenching” process). The specimens were dried under ambient conditions for 2 days and then hot-pressed (Zschokke, Imex Technik AG) using a 1.0-mm-thick spacer, a temperature of 140 °C, and a pressing time of 1 h, resulting in an average thickness of 1.77 mm. Table 1 summarizes the fabrication process details with each sample assigned a specific code.

**Table 1:** Overview of the Fabrication Processes and Corresponding Sample Codes (Del: Delignified, Den: Densified, Rec: Reconstructed)

	delignification at RT (h)	hot delignification (h)	relignification (h)	quenching (h)	hot pressing (h)
Native					
Densified					1
Del-RTD^a^	16				
Del-2HD^a^	16	2			
Del-6HD^a^	16	6			
DenDel-RTD^a^	16				1
DenDel-2HD^a^	16	2			1
DenDel-6HD^a^	16	6			1
Rec-RTD	16		24	1	1
Rec-2HD	16	2	24	1	1
Rec-6HD	16	6	24	1	1

a These samples were analyzed only by XRD and SAXS (Figures S2 and S3).

### 2.4 Characterization

#### 2.4.1 Dry Weight and Lignin Uptake Measurements

To determine their dry weight, the specimens were first oven-dried at 103 °C for 5 h and afterward allowed to cool in a desiccator (over silica gel). To determine the amount of the lignin uptake, the dry weight of the initial specimens was measured and, afterward, the dry weight of the same specimens after the relignification process. The dry weight values were calculated as the averages of five individual measurements.

#### 2.4.2 Scanning Electron Microscopy (SEM)

Specimens were cut perpendicular to the longitudinal direction by using a Trotec Speedy 300 laser cutter (80 W laser power, 1000 Hz frequency). Afterward, they were polished with an ultramicrotome in two sequential steps, first with a cutting glass, then with a diamond knife. To help reveal the microstructure of reconstructed poplar, the surface was polished and, in the case of reconstructed poplar samples, etched with a 15% sodium hypochlorite solution for 60 s to improve the visibility of the microstructure by removing excess lignin from the surface. The specimens were glued to a scanning electron microscope (SEM) stub, and an electrical connection between the surface of interest and the SEM stub was made by using silver ink. Eventually, they were sputter-coated with 5 nm carbon with a Safematic CCU-010 coater. Samples were imaged using a Hitachi SU5000 electron microscope with a 3–5 kV acceleration voltage and a working distance of around 10 mm.

#### 2.4.3 Small-Angle X-ray Scattering (SAXS) and Wide-Angle X-ray Scattering (WAXS)

SAXS and WAXS data were collected using a Xenocs Xeuss 3.0 laboratory SAXS/WAXS system equipped with a Cu K-alpha X-ray source (Genix3D micro source). Samples were placed on a solid sample holder (Xenocs) with the fiber direction perpendicular to the incoming X-rays. The 2D scattering signal was recorded under vacuum on a Dectris EIGER2 1 M detector, positioned at a sample detector distance (SDD) of 400 mm for SAXS and 50 mm for WAXS. The detector distance was calibrated using a silver behenate standard for the 400 mm SDD and lanthanum hexaboride for 50 mm SDD. The beam diameter was set to 0.5 × 0.5 mm^2^ for the SAXS and 1 × 1 mm^2^ for the WAXS measurement. SAXS: The SAXS signals were recorded at three different positions per specimen for 10 min each and afterward averaged. The anisotropic 2D signal was azimuthally integrated along the equatorial plane with an opening angle of 40° to obtain the 1D scattering signal. For the fiber distance determination, the approach presented by Jungnikl et al.^44^ was chosen. The scattering vector, *q*, is calculated according to eq 1 in which 2θ is the scattering angle and λ is the wavelength of the X-rays.

q=4πsin(θ)/λ 1

The distance between the cellulose microfibrils (microfibril distance or interfibrillar correlation length) *d*, which is also called the interplanar spacing or the *d*-spacing, can be calculated from the reciprocal peak position *q*_max_ in the SAXS pattern using eq 2:

$$d=2\pi /q_{\max}$$ 2

To better visualize the peak position, we used the Kratky plot (where *I*(*q*) ∗ *q* ^2^ is plotted over *q*, so emphasizing the peaks). The measurements were conducted also on densified delignified wood as a reference.

WAXS: The WAXS signals were recorded at two different positions per specimen for 5 min each and afterward averaged. The anisotropic 2D signal was azimuthally integrated around 360° and normalized to the cellulose 002 peak.

#### 2.4.4 Water Uptake

Specimens were immersed in deionized water at room temperature up to 80 h, and their thickness was measured on a regular basis. For each specimen, measurements were taken six times at different positions and the values were averaged.

#### 2.4.5 Mechanical Testing

Displacement-controlled tensile experiments were conducted at room temperature and 65% humidity. Specimens were laser-cut at three different fiber directions: along the fibers to investigate axial properties at 0°, off-axis properties at 45°, and transverse properties at 90° relative to the fiber direction, as schematically illustrated in Figure S4. Pieces of beech wood 20 × 15 × 2 mm^3^ (“tabs”) were attached to both ends (15 mm overlap) of the specimens’ length using a Geistlich Holzleim Mirapur 9515 Rapid wood glue. Pictures of the specimens used for the tensile experiment, along with their geometry, are shown in Figure S5. The tensile experiments were carried out with a Zwick-Roell screw-driven universal testing machine. Tests were run with a load cell with a 100 kN capacity and at a constant crosshead speed of 10 mm/min. Strain measurements were performed using a high-resolution video extensometer along the loading direction, where the relative position of two lines marked on the specimen’s surface was measured, and the corresponding strain values were calculated. From the tensile tests, the tensile strength and elastic modulus were determined. The elastic modulus was calculated from a linear portion of the stress–strain curve after removal of the initial nonlinear segment up to a maximum of 40% of the ultimate stress. Four to six specimens were successfully tested for each condition.

#### 2.4.6 Single-Flame Tests

Single-flame tests according to DIN EN ISO 11925–2 were conducted to investigate the ignitability of the composite subjected to direct flame impingement.^45^ In brief, the specimens were first conditioned in a 65% RH climate and then exposed to a flame for 30s. The size of the flame was fixed to 20 mm and oriented in a 45° angle on the surface of the specimen. Deviating from the DIN standard procedure, the time (t120) required for the flame front to reach the upper edge of the sample (120 mm above the flame application point instead of 150 mm) was recorded.

### 2.5 Computational Procedure

Machine learning models were employed to better understand the relationship between the material-fabrication parameters and the mechanical properties of the developed material. By using an unsupervised, supervised classification, supervised regression (USS) learning framework, the influence of material-fabrication process settings (Table S1) on mechanical properties (elastic modulus and strength) was analyzed. Based on these parameters, three material groups were defined: (i) “native poplar”, characterized by zero values for all process parameters and ‘not used’ for hot-pressing-related parameters, (ii) “densified native poplar”, processed solely through hot pressing with varying nominal thickness reduction and temperature, while all other parameters were kept at zero, and (iii) “composites”, fabricated using various combinations of process parameters, representing partially or fully modified poplar structures. The experimental design did not follow a fully exhaustive parameter matrix. Instead, fabrication conditions were iteratively refined based on observed trends of the 0° configuration. Once the optimal parameters were identified for the 0° configuration, additional tests were extended to other fiber directions (45° and 90°). For native poplar and densified poplar, specimens were characterized in all three anatomical directions.

Figure 1a presents the flowchart of the proposed machine learning model. First, the target mechanical properties were clustered into groups using the *k*-means algorithm, which groups the unlabeled data set into different groups based on their similarity.^46^ The algorithm works by randomly picking some central points called centroids, and then each data point is assigned to the closest centroid, forming a cluster. After all of the points are assigned to a cluster, the centroids are updated by finding the average position of the points in each cluster. This process is repeated until the centroids no longer change. To determine the optimal number of clusters, the elbow method was applied, in which a range of *k* values, typically from 1 to *n* (where *n* is a chosen hyperparameter), is iterated over.^46^ For each *k*, the within-cluster sum of squares (WCSS) is calculated. The WCSS quantifies how accurately the data points around their respective centroids are clustered and is defined as the sum of the squared distances between each point and its cluster centroid. The optimal *k*, elbow point, is identified where adding more clusters yields only marginal improvements in the clustering performance.

**Figure 1:**
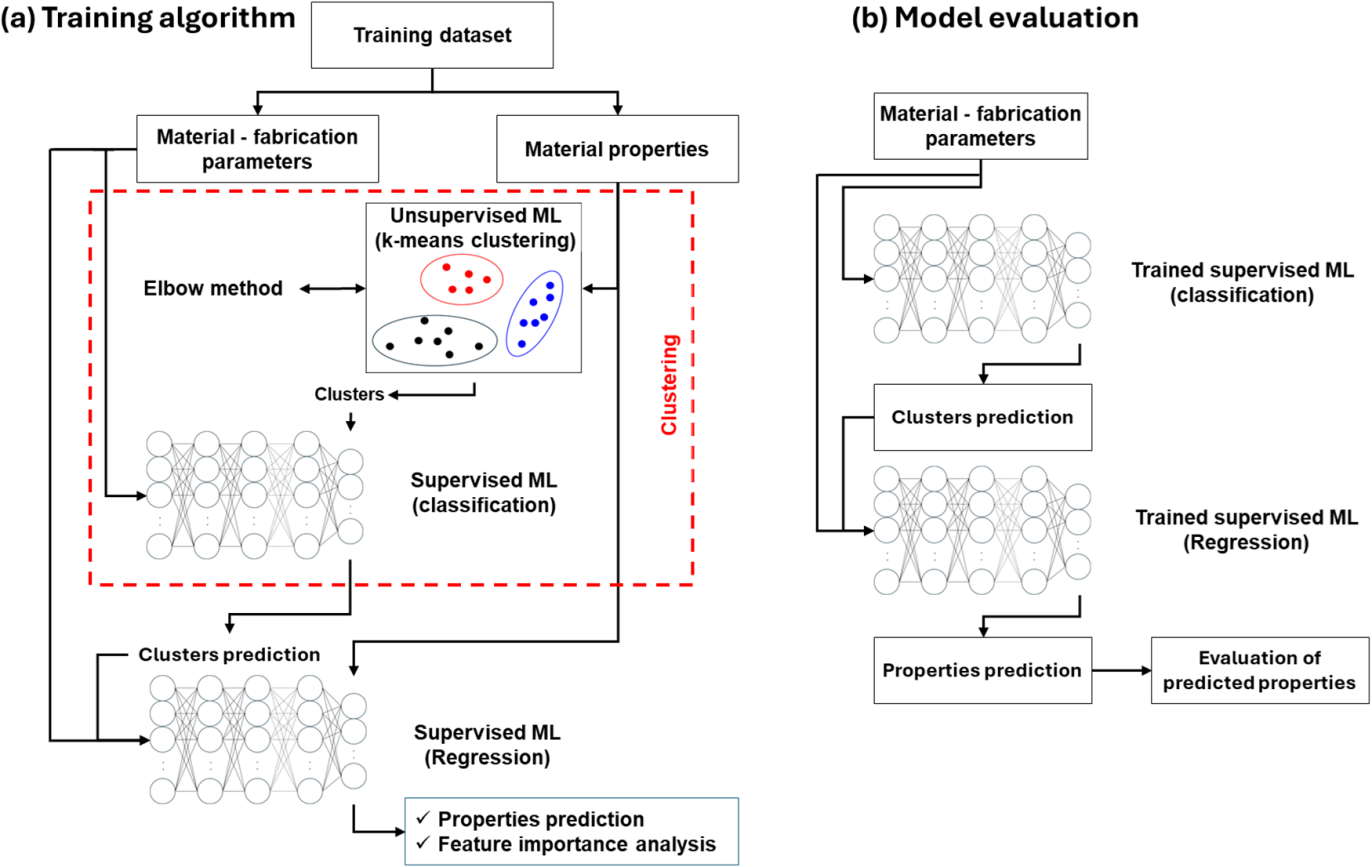
(a) Schematic representation of the ‘unsupervised, supervised classification, supervised regression’ (USS) learning framework used in this work, consisting of a *k*-mean clustering step of material properties, a supervised classification model in which the input is material-fabrication parameters and targets their corresponding clusters, and a supervised regression model in which material-fabrication parameters as well as the predicted clusters are the input to predict the material properties. (b) Flowchart for model evaluation in which a trained supervised classification model is first used to cluster the fabrication parameters, followed by a supervised regression model that predicts the mechanical properties, based on both the material-fabrication parameters and the assigned clusters.

Afterward, the material-fabrication parameters were used as input features to train a classifier fully connected neural network (FCNN). FCNNs are a type of artificial neural networks (ANNs) characterized by layers of interconnected neurons where each neuron in one layer is connected to every neuron in the next. FCNNs are well-suited for modeling complex relationships and patterns within data sets by processing information through multiple layers.^42^ A common architecture within FCNNs is the multilayer perceptron (MLP), which includes an input layer, one or more hidden layers, and an output layer.^46^ In this work, the model was trained via forward propagation, backpropagation, and gradient descent-based optimization, and to mitigate the impact of class imbalance during training, class weighting (*w*_i_) was applied according to eq A3. The cluster labels from the previous step served as the target outputs, forming a self-supervised learning model. This approach enabled the model to learn the correlation between the fabrication process parameters and clusters. To estimate the actual mechanical performance, an FCNN regressor was employed. The model used both the material-fabrication parameters and the predicted cluster labels, obtained from the classifier FCNN, as input features, while the outputs were continuous predictions of the elastic modulus and tensile strength. After training the (USS) learning framework, permutation-based feature importance analysis^47,48^ was applied to regressor FCNNs to identify the most influential fabrication parameters contributing to the predicted mechanical properties.

In this study, a data set comprising around 230 entries was compiled, each containing material-fabrication parameters and their corresponding mechanical properties. Before model development, all numeric variables, including material-fabrication parameters (Table 1), were standardized while the mechanical properties (elastic modulus and strength) were log-transformed prior to standardization, to ensure that each predictor contributes equally to the training process. Figure S7 shows the range of the standardized material-fabrication parameters and properties. To prevent data leakage, all repetitions of a given condition were grouped and assigned entirely to either the training or the test set. In total, 80% of the data was used for training and 20% for testing. Additionally, to ensure reliable performance assessment, the network was trained using a 5-fold cross-validation approach (Appendix A.2), where 20% of the training data set in each fold was used for validation. The classifier FCNN was designed with three hidden layers containing 64, 32, and 8 neurons using ReLU activation in the hidden layers and softmax in the output layer. It was trained with a batch size of 64, a learning rate of 0.001, a regularization parameter of 0.03, and an Adam optimizer. Sparse Categorical Crossentropy was used as the loss function, and early stopping was applied with a patience of 5 epochs. Class weighting was applied as C1 = 0.678, C2 = 1.264, C3 = 0.866, and C4 = 1.731. The regressor FCNN comprised four hidden layers with 256, 128, 64, and 32 neurons, all using ReLU activation, except for a linear output layer. It was trained with a batch size of 128, a learning rate of 0.001, and an Adam optimizer. The mean squared error (MSE, eq A2) served as the loss function, and early stopping was applied with a patience of 100 epochs. Table S2 also summarizes the architecture of the regressor FCNN as well as the other used hyperparameters.

Figure 1b illustrates the workflow used to evaluate the performance of the trained model. First, the material-fabrication process parameters are inserted into the trained unsupervised machine learning model to predict the corresponding cluster. This predicted cluster and the original material-fabrication parameters are then fed into the trained supervised model to predict the mechanical properties. Model performance was assessed by comparing the predicted properties with experimental values for both training and testing data sets and quantified using the coefficient of determination *R* ^2^ (see Section A.5).

### 2.6 Life Cycle Assessment (LCA)

A life cycle assessment (LCA) was conducted in accordance with ISO 14040 to compare the environmental impacts of reconstructed poplar through room-temperature delignification and reusing the RT delignification solution. A cradle-to-gate scope was used. The inventory analysis was conducted using the Ecoinvent 3.10 database.^49^ If the input was not found in the database, the impacts were modeled based on processes described in the literature or analogous materials. Details can be found in the Supporting Information section. We performed the impact assessment using the ReCiPe 2016 ^50^ methodology, focusing on the global warming potential (GWP) midpoint category and ecosystem quality (EQ) endpoint category. To perform the LCA, Activity-Browser software was used. A scale-up framework developed by Piccinno et al. was implemented for all cases.^51^ As a functional unit, kilogram of composite production was used

## 3 Results and Discussion

### 3.1 Poplar Wood Reconstruction

Figure 2a shows the implemented approach, starting from (i) structural-retaining delignification (from extensive to partial), followed by (ii) relignification, i.e., impregnation with an aqueous solution of Kraft lignin sodium salt (“water-soluble lignin”), and finally (iii) densification, resulting in “reconstructed poplar” featuring a strong improvement of mechanical properties together with high water stability.

**Figure 2:**
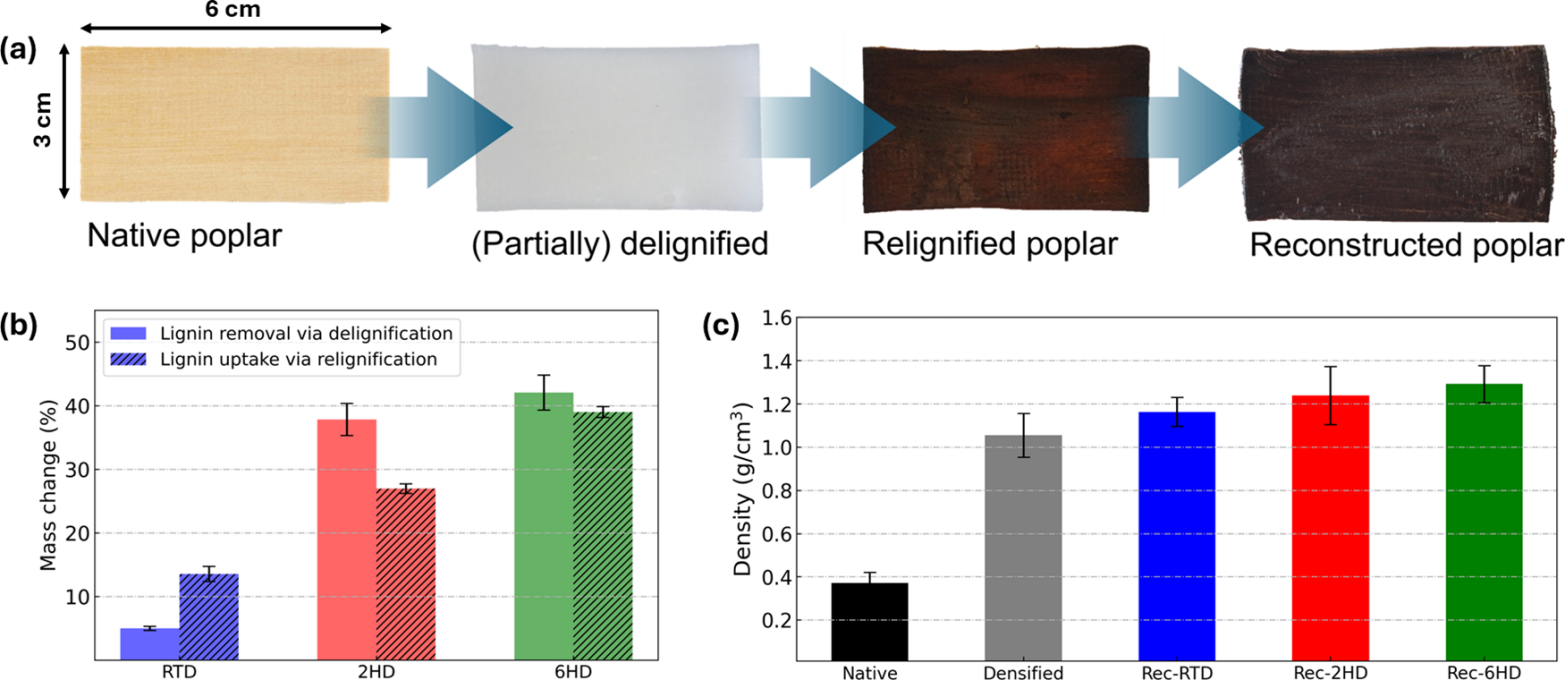
(a) Fabrication process of reconstructing poplar. Native, delignified, relignified, and reconstructed poplar and (b) lignin removal through delignification and lignin uptake via relignification under different conditions. (c) Density of native, densified native, and reconstructed poplars: Rec-RTD, Rec-2HD, and Rec-6HD.

Native poplar was delignified using the acetic acid–hydrogen peroxide method under various conditions, resulting in different levels of lignin (and hemicellulose) removal. As shown by gravimetric analysis (Figure 2b), the mass loss was 5% after 16 h room-temperature delignification and increased considerably (to 42%) after 6 h at 80 °C. Compared to the samples produced via room-temperature delignification, those delignified at high temperature were exceedingly fragile due to the significant lignin removal. The delignified wood samples were relignified by impregnating them with a water-soluble sodium salt of Kraft lignin (“soluble lignin”) under ambient conditions for 24 h, leading to dark-brown colored samples. In principle, the relignification procedure could be performed by using lignin dissolved in sodium hydroxide. The main advantages of soluble lignin include a readiness of dissolution in water, a milder pH of the resulting solution (ca. 8), and a much lower tendency to foam upon stirring. The lignin uptake for the specimens delignified at room temperature led to a 13% lignin uptake compared to 39% for specimens that were hot-delignified for 6 h. The samples were quenched in an aqueous citric acid solution, causing lignin to precipitate and hydrophobize the composite. Afterward, the samples were removed from the solution, and after they had reached moisture equilibration at 20 °C under 35% RH, they were hot-pressed at 140 °C for 1 h, yielding reconstructed poplar. Native and delignified poplar were also densified under the same hot-pressing conditions to serve as reference samples.

### 3.2 Microstructural Characterization

The density variation for the specimens investigated is shown in Figure 2c. Native poplar exhibited an average density of 0.39 g cm^–3^, while densified native poplar reached 1.05 g cm^–3^. For specimens that were delignified at room temperature, the average density of densified relignified specimens further increased to about 1.1 g cm^–3^. The longer the time of hot delignification, the higher the density of the reconstructed poplar, reaching more than 1.2 g cm^–3^ for the 2 h hot-delignified sample and 1.3 g cm^–3^ for the 6 h hot-delignified sample.

Scanning electron microscopy (SEM) was used to investigate the effect of delignification on the microstructure of reconstructed poplar compared with that of native and densified native poplar. Figure 3a shows a typical native poplar microstructure. As shown in Figure 3b, the cell walls are considerably deformed after densification. The microstructure of the reconstructed poplar, delignified at room temperature (Figure 3c), reveals significantly deformed cell walls and filled lumina (most likely with precipitated lignin). As the hot delignification time increased, e.g., after 2 h (Figure 3d), the cell walls became severely deformed. Hot delignification for 6 h led to completely collapsed cells (Figure 3e) compared with native poplar and partially delignified poplar, which indicates an increased deformability of the cell walls, resulting in a more compact structure. To help localize the impregnating lignin, energy-dispersive X-ray spectroscopy (EDX) was performed on a representative reconstructed poplar sample after selective labeling with bromine (Figure S1).^52^

**Figure 3:**
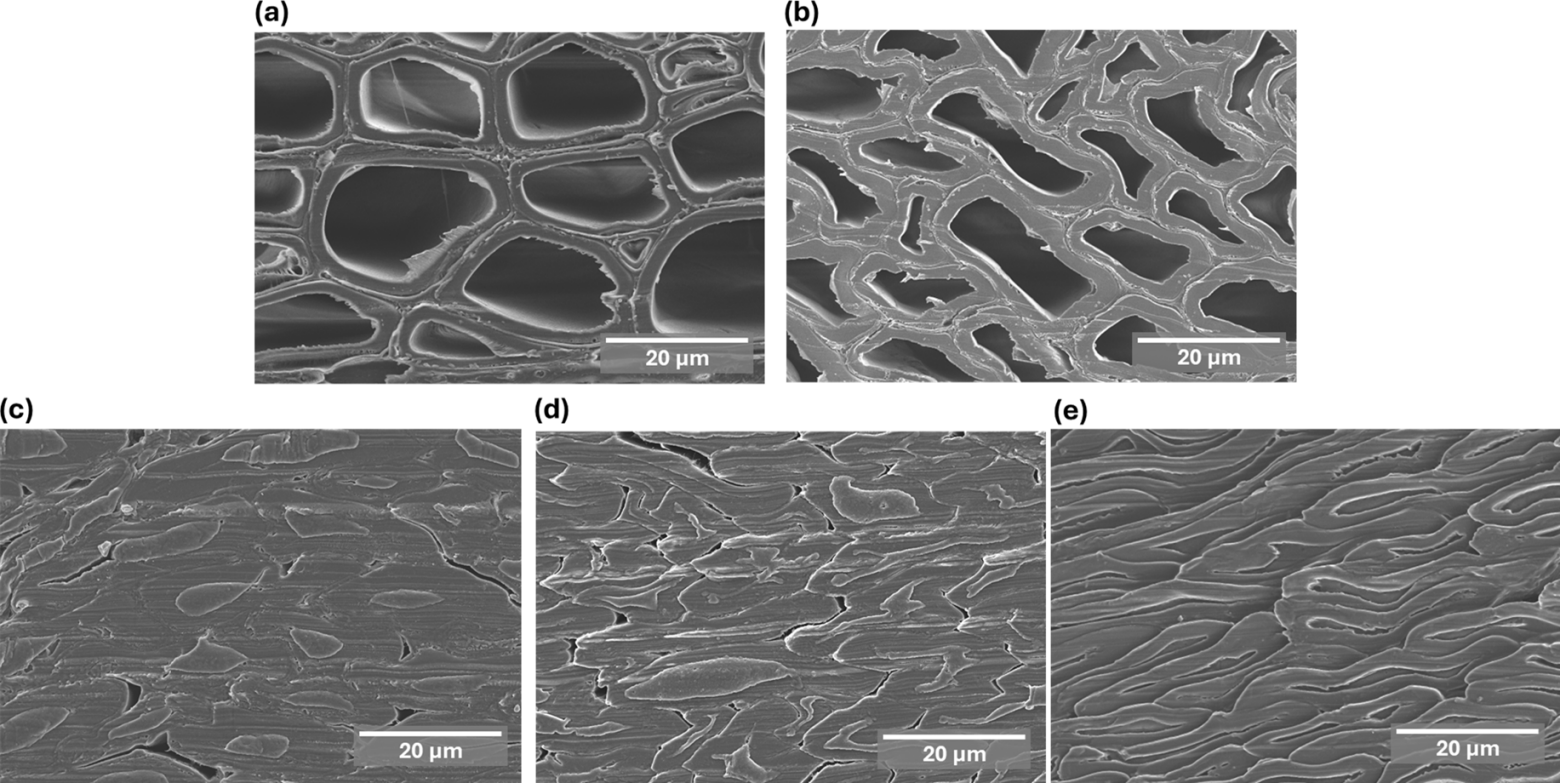
SEM images showing the microstructure of (a) native poplar; (b) densified native poplar; and reconstructed poplar under delignification levels: (c) Rec-RTD, (d) Rec-2HD, and (e) Rec-6HD.

To evaluate the effects of different delignification levels on the nanostructural features of poplar, wide-angle X-ray scattering (WAXS) and small-angle X-ray scattering (SAXS) analyses were performed and are shown in Figure S2a–f. Three sample types, namely, delignified poplar (DelPop), densified delignified poplar (DenDelPop), and reconstructed poplar (RecPop), were examined (Table 1). In all cases, three peaks were detected, corresponding to the 101, 002, and 040 planes of cellulose I. The data was normalized to the 002 peak and a reduction in background intensity was observed from native to fully delignified poplar, suggesting a decrease of the amorphous scattering signal. This could indicate an increase in crystallinity resulting from lignin removal. The average distance between cellulose microfibrils was determined using small-angle X-ray scattering (SAXS) and is presented in Figure S3. A shift of the scattering peak toward higher *q*-values for samples delignified for more than 2 h indicates a reduction in fiber spacing due to delignification. Prolonged delignification beyond this point does not lead to significant further changes in fiber distance. Notably, the subsequent densification process did not affect the fiber spacing.

### 3.3 Mechanical Properties

To evaluate the mechanical properties of the reconstructed poplar fabricated under different conditions, tensile tests were conducted at three fiber directions of 0°, 45°, and 90° relative to the loading direction. Representative stress–strain curves of native wood, densified wood, Rec-RTD, Rec-2HD, and Rec-6HD specimens in tension with 0°, 45°, and 90° fiber directions are shown in Figure S6. The elastic modulus values of reconstructed poplar samples were compared to those of native and densified native poplar, as shown in Figure 4a. It was observed that densification resulted in average 0° elastic modulus values approximately double compared to that of native poplar. The elastic modulus increased further for Rec-RTD, averaging 23 GPa, with the highest elastic modulus observed for Rec-6HD, reaching 33 GPa. Similar to the 0° elastic modulus, both densification and reconstruction enhanced the 45° and 90° elastic moduli. Nevertheless, all of these values decreased significantly compared to those measured for the 0° elastic modulus. In addition, the elastic moduli measured for reconstructed poplar at 45° fiber directions were nearly independent of the type of the implemented delignification process, averaging around 6 GPa. At 90° however, a slight difference was observed in the elastic modulus of Rec-6HD (approximately 5.5 GPa) compared with Rec-RTD and Rec-2HD (around 4 GPa). Nonetheless, for both fiber directions, the values remained higher compared to the densified native wood. The specific values of the elastic modulus are shown in Figure 4b. The investigated materials displayed comparable longitudinal specific elastic modulus values, implying that densification was the main factor of this property improvement. By contrast, reconstructed poplar samples displayed higher 45° and 90° specific elastic modulus values, suggesting that other mechanisms, in addition to densification, were responsible for the observed strengthening.

**Figure 4:**
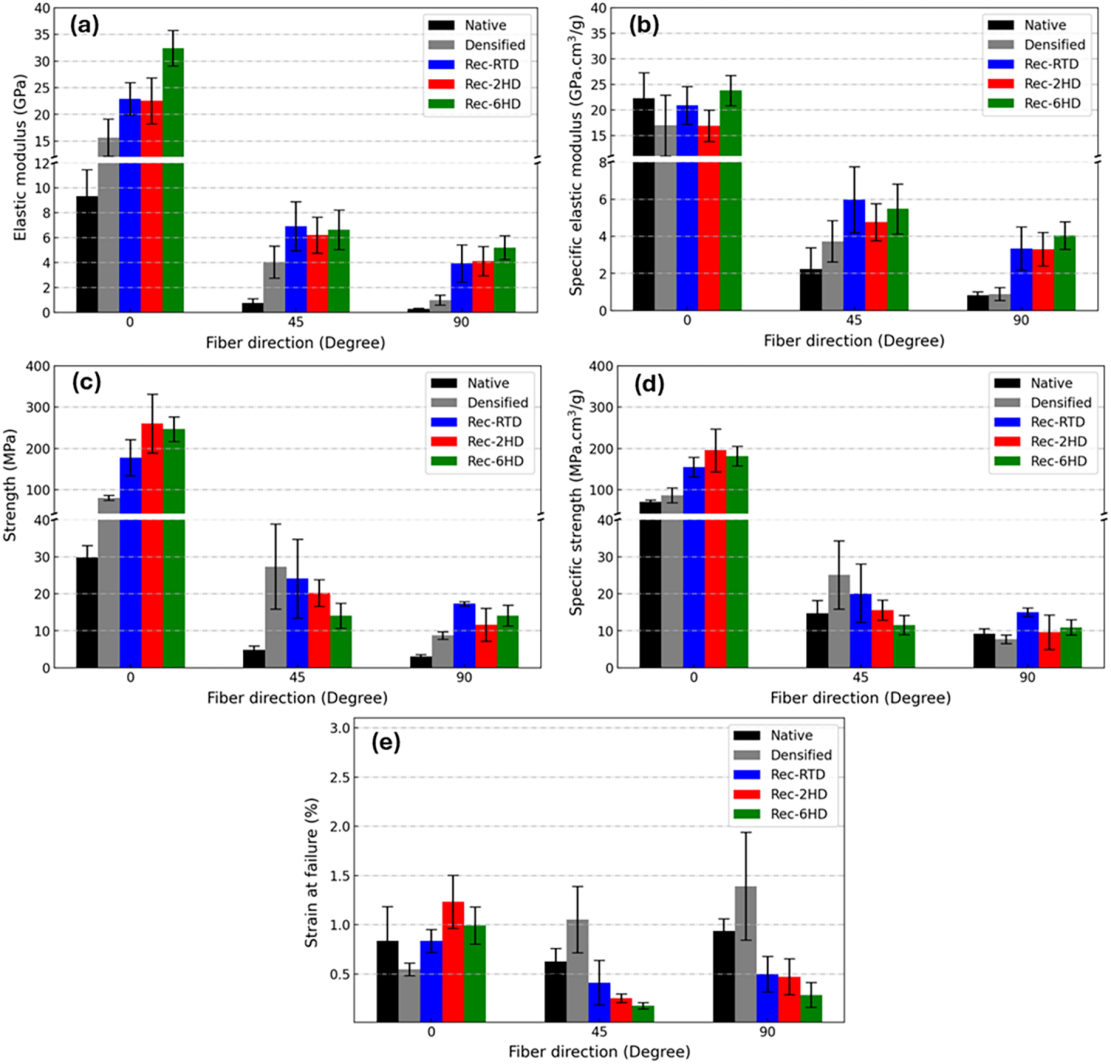
Comparison of the mechanical properties of native poplar, densified native poplar, and reconstructed poplars: Rec-RTD, Rec-2HD, and Rec-6HD. (a) Elastic modulus, (b) specific elastic modulus, (c) strength, and (d) specific strength. (e) Comparison of the strain at failure in native poplar, densified native poplar, and reconstructed poplars: Rec-RTD, Rec-2HD, and Rec-6HD.

The strength values and their specific values are shown in Figure 4c,d, respectively. Reconstructed poplar wood obtained from room-temperature delignification (Rec-RTD) displayed a high 0 °C average strength value of 178 MPa (with a specific value of 155 MPa), while samples that were hot-delignified for 2 and 6 h reached remarkably high values of 261 and 247 MPa, respectively, (with specific values of 194 and 181 MPa). These values were about eight times and three times higher compared to native and densified native poplar. It is noteworthy that the composites obtained by hot delignification displayed an increase not only in their strength values but also in their specific strength values. This result cannot solely be explained as an effect of densification, but it becomes understandable taking into account the higher lignin uptake of these samples during relignification (Figure 2b), as the resulting enhanced interfacial bonding between adjacent cell walls would have led to an improved structural integrity.

Unlike the 0° results, the hot delignification process decreased the 45° strength value of the reconstructed poplar from 24 MPa (Rec-RTD, with a specific value of 20 MPa) to 14 MPa (Rec-6HD, with a specific value of 12 MPa) on average. In the 90° direction, it was observed that the strength and specific strength values increased in all reconstructed poplar samples compared to native and densified native poplar. Nevertheless, the highest strength value in the 90° direction was obtained for specimens with room-temperature delignification. The strain at failure for the different specimens at the three reorientation angles is summarized in Figure 4e, showing that the reconstructed poplar prepared by hot delignification had a lower strain at failure at 45° and 90° fiber directions. It is due to the materials' brittleness, which reduced its ability to sustain off-axis loads, leading to lower strength values.

### 3.4 Water and Fire Resistance Behavior

Figure 5a shows the water resistance behavior of native poplar, densified native poplar, and reconstructed poplar samples in the form of thickness evolution as a function of immersion time in water. Native poplar showed a thickness increase of 6%, while densified native poplar exhibited a dramatic spring-back effect, almost completely recovering its predensification thickness. The water resistance was remarkably improved in the case of reconstructed poplar samples, as evidenced by a significant reduction in the thickness increase. The delignification process softened the cell walls, causing them to collapse during the hot pressing and resulting in a wood material compaction. Additionally, during hot pressing, lignin was plasticized and acted as a bonding agent, adhering to and enveloping the internal lumen walls (as illustrated in Figure 3 for reconstructed wood). Figure 5 also demonstrates that hot delignification further enhanced the water stability of the reconstructed poplar. The use of hot delignification led to a greater degree of lignin removal, facilitating the uptake of lignin during relignification (Figure 5b), and allowed a more efficient collapse of the cells during densification, resulting in a tighter structure^28,53^ with a higher density (Figure 5c). The Rec-6HD sample demonstrated excellent water resistance, exhibiting only a minimal thickness increase, even after prolonged immersion. Figure 5b shows the percentage of thickness increase for all tested samples after 80 h immersion in the water, showing that while densified wood exhibited a dramatic spring-back effect, the behavior of Rec-6HD was slightly higher than that of native wood. Rec-RTD was the least water-stable among the three reconstructed poplar samples.

**Figure 5:**
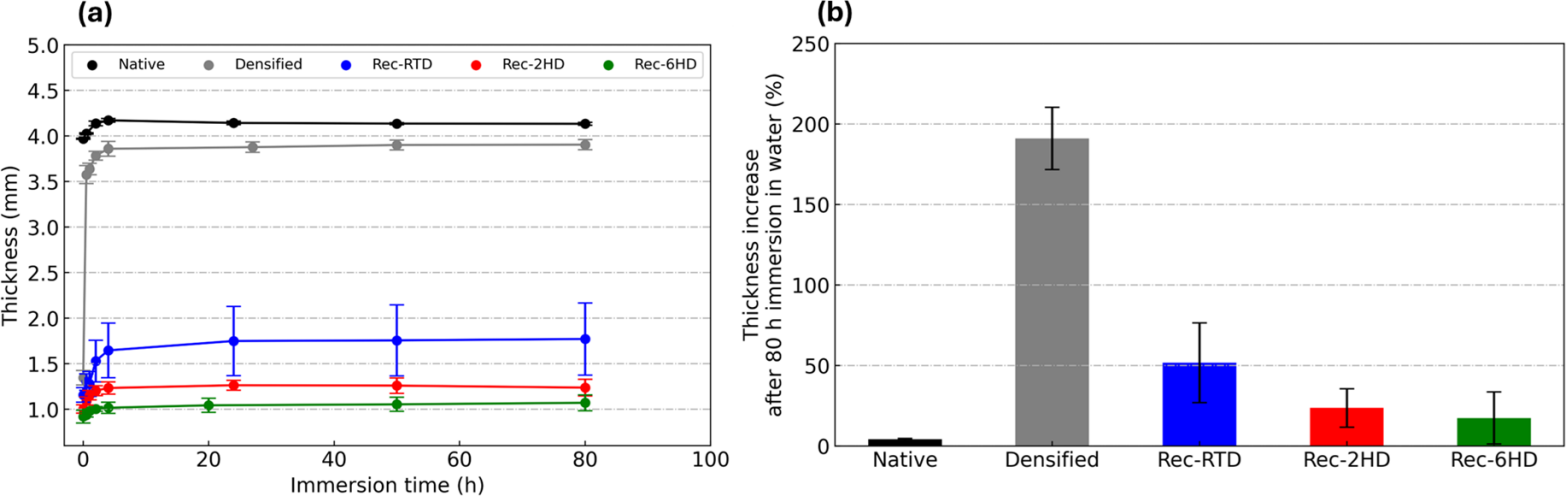
(a) Variation of thickness at different immersions and (b) time thickness increase after 80 h of immersion in water for native poplar, densified native poplar, and reconstructed poplar (Rec-RTD, Rec-2HD, and Rec-6HD).

To gain initial insights into the fire resistance of native poplar, densified native poplar, and Rec-RTD samples, single-flame experiments were conducted. The results of single-flame experiments are shown in Figure 6. It was observed that Rec-RTD displayed enhanced fire resistance,as evidenced by a significantly smaller burnt surface as well as the absence of fire ignition events, in contrast to the native and densified native poplar. The improved flame behavior of the reconstructed samples could reasonably result from a combination of their higher content of char-forming lignin and their more compact structure, limiting oxygen diffusion. Nonetheless, further tests at larger scales are required, as the single-flame experiment only provides a first and qualitative performance indication.^54^

**Figure 6:**
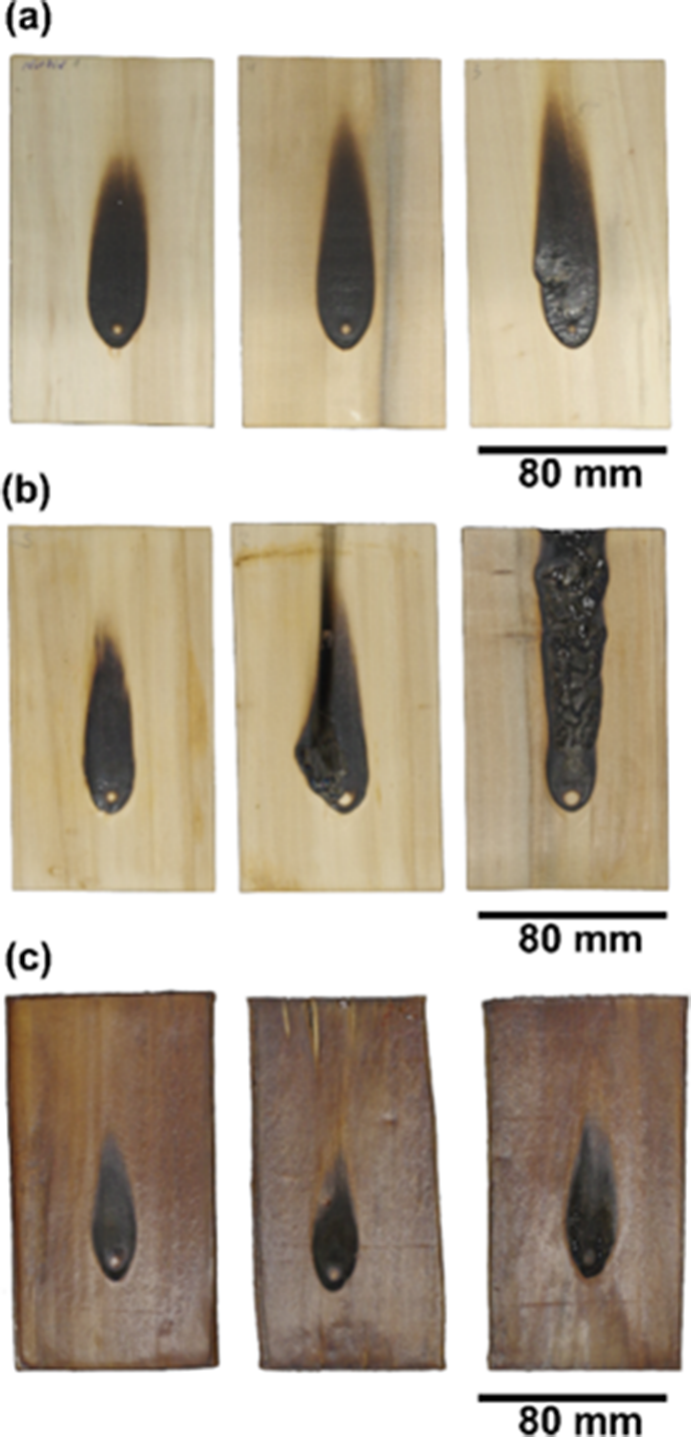
Fire resistance test (single-flame exposure, three repetitions) for (a) native poplar, (b) densified native poplar, and (c) Rec-RTD.

### 3.5 Machine Learning-Based Analysis for Property Prediction

Figure 7a shows the variation of the WCSS across different cluster numbers. As the number of clusters increases, the WCSS decreases sharply up to 4 clusters, after which the decrease becomes more gradual, indicating that 4 is the optimal number of clusters (see Section 2.5). To better visualize the clustering, a 3D plot is shown in Figure 7b, depicting strength as a function of the elastic modulus and density for the 4 identified clusters.

**Figure 7:**
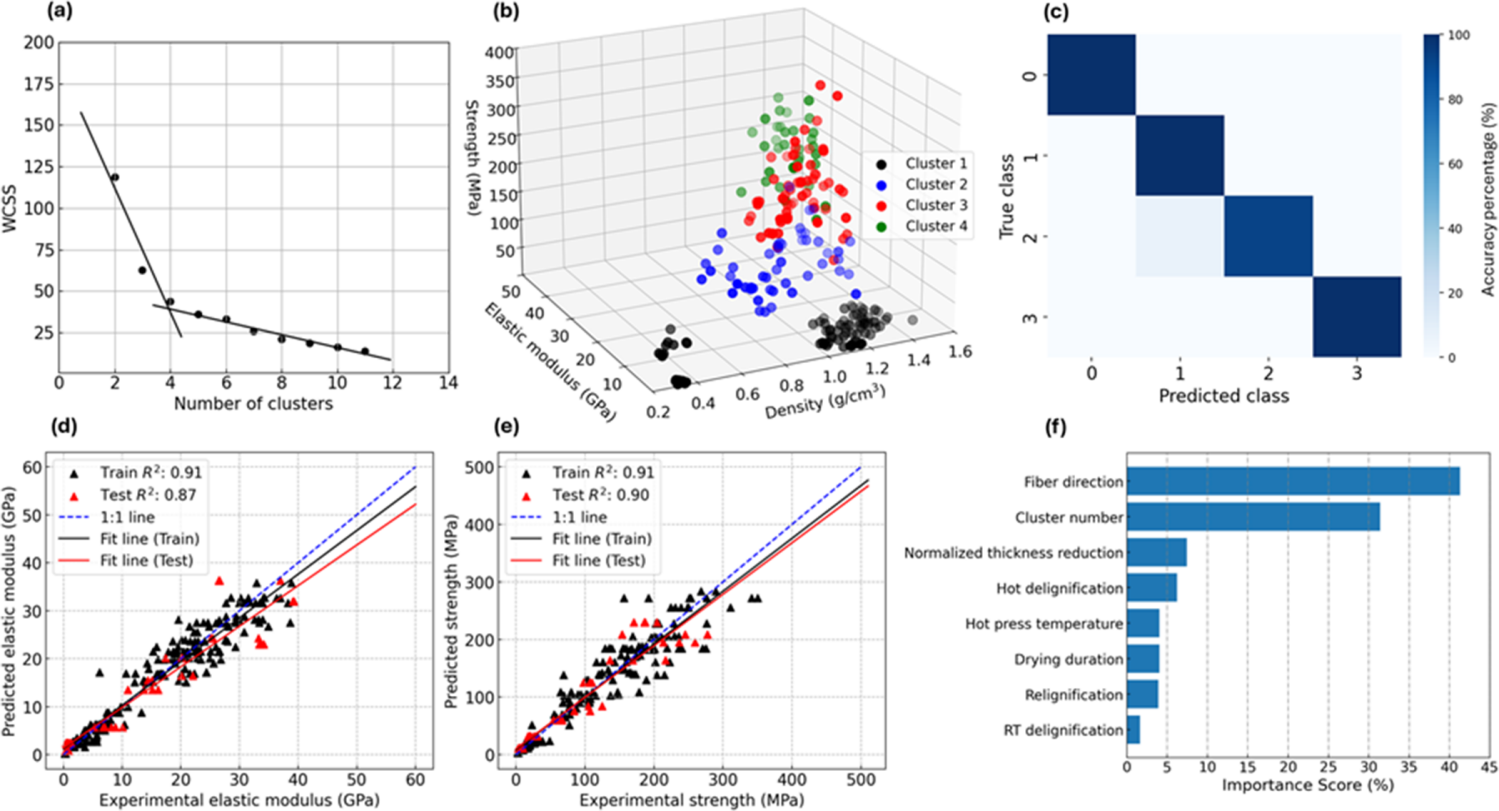
(a) Variation of WCSS with respect to the number of clusters. (b) 3D illustration of strength–elastic modulus–density for the 4 identified clusters. (c) Confusion matrix showing the correspondence between predicted and true clusters and correlation between predicted and experimental values for (d) elastic modulus and (e) strength, using the ‘unsupervised, supervised classification, supervised regression’ (USS) learning framework. (f) Feature importance analysis using the permutation method.

The architectures of the classifier FCNN as well as the other hyperparameters are summarized in Table S2. After the training, the performance of the FCNN was evaluated by comparing the predicted class with the true class, as shown in Figure 7c. It is seen that the model was capable of properly predicting the correct class, as shown using a confusion matrix, leading to an F1-average of 0.98. The material-fabrication parameters together with their corresponding predicted clusters were imported as input for a regressor FCNN to predict their elastic modulus and strength. Figure 7d,e demonstrates the predicted elastic modulus and strength values with respect to their counterpart experiments. In both cases, the 1:1 line serves as a reference for perfect agreement between prediction and the experiment, while regression lines indicate the best fit between predicted and experimental values in the training and testing data sets. It can be observed that *R* ^2^ scores for the elastic modulus and strength predictions on the training data set are both 0.91, indicating that the input neural network model has been properly trained. Furthermore, high *R* ^2^ scores of 0.87 and 0.90 in predicting the elastic modulus and strength in the testing data set proved that the trained model was performing well without any overfitting. A 5-fold cross-validation was performed to assess the model robustness for generalization, and the results are summarized in Table S3. The classifier FCNN achieved an F1 score of 0.979 ± 0.034 on the training set and 0.963 ± 0.040 on the validation set, while the regressor FCNN achieved an average *R* ^2^ of 0.941 ± 0.019 on the training set and 0.919 ± 0.034 on the validation set. These results indicated the model’s good generalization within the range of fabrication parameters included in the training data. Due to the high performance of the regressor FCNN in predicting mechanical properties, the use of the trained model in feature importance analysis can be considered to be reliable for determining the contribution level of each feature in training the model. Permutation-based feature importance, shown in Figure 7f, revealed that fiber direction and cluster number were the most significant features. The prominence of fiber direction reflected the anisotropic nature of the composite, while the cluster number, obtained through the unsupervised–supervised ML approach, enabled effective classification of the elastic modulus and strength, contributing to improved prediction accuracy. Nominal thickness reduction was the most influential fabrication parameter for predicting the mechanical performance of the reconstructed wood. This underscores the significant role of densification in determining the final compaction level of the material, which strongly correlates with mechanical strength. Hot delignification was also considered one of the most important fabrication parameters, mainly responsible for the extent of lignin removal and subsequent lignin uptake during relignification (see Figure 2c).

The predicted results using the USS model were also compared with those obtained by using a simple End-to-End FCNN (Figure S8), in which the mechanical properties were predicted using material-fabrication parameters in Figure S9. Both models perform similarly on the training data, indicating comparable learning capacity; however, the USS framework achieved higher accuracy on the test data, because the initial clustering step allowed the model to better distinguish patterns within the data, leading to improved generalization.

### 3.6 Life Cycle Assessment (LCA) and Up-Scaling

One important aspect of poplar reconstruction using the room-temperature delignification process is that it allows reuse of the delignification solution. This is because unlike high temperature delignification, where the decomposition of hydrogen peroxide is accelerated, at room temperature, its concentration should remain more stable over time. Of course, this also results in a lower delignification efficiency. Figure 8a shows the variation in density and strength of reconstructed poplar as a function of the number of times the delignification solution was reused: once (Rec-RTD1), twice (Rec-RTD2), and three times (Rec-RTD3). The results indicate that the density and strength of Rec-RTD were almost independent of the number of times the delignification solution was reused. The possibility to reuse several times the delignification solution without compromising the mechanical properties of the final composite suggests an increased sustainability of the process.

**Figure 8:**
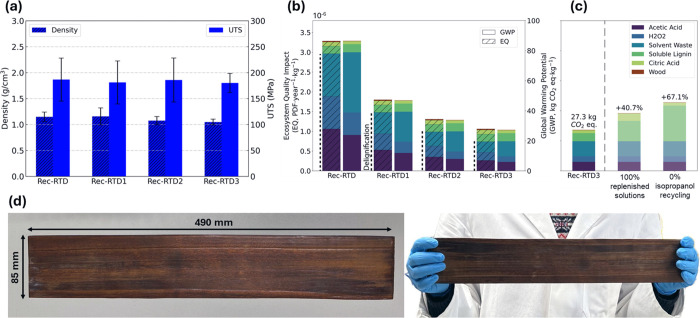
(a) Density and strength of reconstructed poplar delignified at room temperature, Rec-RTD, versus the number of uses of delignification solution. (b) Global warming potential (GWP) and ecosystem quality (EQ) impacts for samples 6HD Rec-RTD, Rec-RTD1, Rec-RTD2, and Rec-RTD3. (c) Sensitivity analysis of Rec-RTD3, assessing the impact of the distillation assumption for isopropanol in the soluble lignin processing (no dist.) and ±20% variation per kg of reconstructed poplar. (d) Example of the up-scaled Rec-RTD with the dimension of 490 × 85 mm^2^.

A flow diagram with system boundaries for the different options considered in this work for life cycle assessment (LCA) is shown in Figure S10. LCA demonstrated that the global warming potential (GWP) and ecosystem quality (EQ) impacts per kg of reconstructed poplar were predominantly driven by the delignification process, as shown in Figure 8b, on all room-temperature delignified (RTD) samples using an upgraded framework (see Section B in Appendixes for details). The LCA results demonstrate that the repeated reuse of the delignification solution effectively reduces the environmental impact of reconstructed poplar, even though it is obviously higher compared to that of native poplar wood.

To assess the robustness of these findings, a sensitivity analysis was performed on two key assumptions (Figure 8c). In the Rec-RTD3 base scenario, it was assumed that 80% of both lignin and citric acid solutions were reused (so that only 20% of the lignin and citric acid solutions would need to be replenished using primary materials; see the SI for details) and that the isopropanol used for lignin dissolution was recycled. By contrast, using 100% replenished solutions would lead to a 40.7% increase in GWP, and removing the isopropanol recycling assumption would result in an even higher (67.1%) increase in GWP. Accordingly, we can see that future work should prioritize reducing the impacts associated with the delignification solution and the production of soluble lignin.

To demonstrate the up-scaling potential of our wood reconstruction method, we produced a 490 × 85 mm laboratory-scale demonstrator of Rec-RTD, as shown in Figure 8d. To ensure an effective process, the delignification time was extended to 48 h, followed by relignification for an additional 48 h, allowing for thorough lignin removal and reintegration. These adjustments were made to accommodate the increased sample size and ensure a uniform treatment throughout the material.

## 4 Conclusions

In this work, we demonstrated that the process of poplar reconstruction into a high-performance biobased fiber-reinforced composite by performing partial delignification at room temperature yielded significantly improvements, in terms of both up-scaling potential and environmental sustainability aspects relative to fully delignified poplar. Additionally, we observed that reconstructed poplar fabricated via room-temperature delignification displayed superior mechanical properties at 45° and 90° fiber directions compared to native poplar, densified native poplar, and fully delignified poplar, thereby expanding its possible range of applications, e.g., as a construction material, where high strength across multiple orientations is required. However, these improvements came at the cost of lower mechanical properties in the 0° direction and an increased swelling after water immersion, compared to samples fabricated with high-temperature full delignification, underscoring a trade-off that should be taken into account application-wise. The use of a machine learning model comprising a ‘unsupervised, supervised classification, supervised regression’ (USS) learning framework allowed us to predict well the properties that could be obtained with different material-fabrication parameters. Furthermore, the feature importance analysis offered a data-driven justification for identifying the fiber direction, cluster number, and nominal thickness reduction as the key parameters governing the mechanical performance of the reconstructed poplar. Lastly, performing room-temperature delignification allows for reusing several times the same delignification mixture without compromising the material properties. As shown by life cycle assessment (LCA), this reduces the environmental impact of the process and minimizes the global warming potential (GWP) per kilogram of composite production.

Future work should focus on enhancing on-axis properties, improving water stability, conducting further fire resistance tests, and up-scaling to a demonstration level and finally to an industrial scale. Expanding the data set to include other wood species could further improve the generalizability of the predictive USS framework.

## Supplementary Material



## A Machine Learning-Based Analysis for Property Prediction

### A.1 Data Standardization

The target values were transformed into a logarithmic scale and, together with the material-fabrication parameters, were standardized prior to the simulation. To standardize a dataset, eq A1 is applied, where *X* represents the original data point, μ is the mean, and σ is the standard deviation of the dataset. This process rescales the data to have zero mean and unit variance, resulting in standardized values that improve comparability across features.

$$X_{\mathrm{standardized}}=\frac{X-\mu }{\sigma }$$
A1

### A.2 Cross-Validation

When searching for the best estimator, there is always a risk of overfitting during the learning process, which reduces the model’s generalizability and lowers prediction accuracy. To prevent overfitting, a procedure called cross-validation is commonly used. In *k*-fold cross-validation, the training set is split into *k* smaller subsets (folds). The model is trained on *k*-1 folds and validated on the remaining fold. This process is repeated *k* times, each time using a different fold as the validation set, and the final performance is computed as the average across all k iterations.

### A.3 Mean Squared Error

Mean squared error (MSE) is a measure of the average squared difference between the actual values (*y*_exp_) and the predicted values (*y*_pre_) in a dataset. It is defined as

$$\mathrm{MSE}=\frac{1}{n}\sum_{i=1}^{N}(y_{\exp,i}-y_{\mathrm{pre},i}{)}^{2}$$
A2

in which *n* is the total number of data points.

### A.4 Class Weighting for Imbalanced Data Handling

To mitigate the impact of class imbalance during training, class weighting (*w*_i_) was applied. This approach assigns higher weights to underrepresented classes and lower weights to overrepresented ones, ensuring that the model does not become biased toward the majority class. The weights were computed according to the inverse frequency of each class:

$$w_{i}=\frac{n_{\mathrm{samples}}}{n_{\mathrm{classes}}\cdot n_{i}}$$
A3

where *n*_samples_ denotes the total number of samples, *n*_classes_ denotes the number of samples in class *j*, and *nj* is the total number of classes. These weights were integrated into the loss function to emphasize minority classes and promote balanced learning across all categories.

### A.5 Coefficient of Determination

The prediction accuracy was assessed by using the coefficient of determination, *R* ^2^, calculated according to eq A4:

$$R^{2}=1-\frac{\overset{N}{\underset{i=1}{\sum }}{\left(y_{\exp}-y_{\mathrm{pre}}\right)}^{2}}{\overset{N}{\underset{i=1}{\sum }}{\left(y_{\exp}-y_{\mathrm{mean}}\right)}^{2}}$$
A4

The coefficient varies in a range of 0.0, for the case that the model does not predict accurately, to 1.0, for a fully accurate prediction, where *y*_mean_ stands for the mean experimental value.

## B Life Cycle Assessment

For the life cycle assessment (LCA) of reconstructed wood, the assumptions, calculations, and data sources used to calculate environmental impact are described in detail. A cradle-to-gate analysis was performed, using the Ecoinvent 3.10 database.

### B.1 Scope and Functional Unit

The LCA scope is cradle-to-gate, excluding the use and end-of-life phases. The functional unit is defined as the environmental impact per kilogram of composite production. This unit was selected to enable comparability with other materials.

### B.2 Up-Scaling Assumptions

The environmental impact of a developed material is largely influenced by its manufacturing and use once scaled-up. Therefore, assessing the impact on an industrial scale is essential. To scale up reconstructed wood from lab-stage data, we applied the scale-up framework developed by Piccinno et al. for chemical processes.^51^ This approach required several key assumptions:

- Heating: Conducted in 1000 L insulated reactor tanks.
- Solvent Reduction: A 20% reduction was assumed for all solvents.
- Solvent Recycling: Solvents were assumed to be recycled, accounting for energy use in distillation, waste generation, and distillation yield. This was particularly critical for isopropanol recycling, which also requires water content reduction using salts. To evaluate this assumption’s impact, a sensitivity analysis was performed with and without solvent recycling (see Table S9).
- Geographic Location: All inputs were assumed to originate in Europe (RER or Europe without Switzerland in the Ecoinvent 3.10 database).
- Infrastructure: Extrapolated based on data from the Ecoinvent 3.10 database, assuming a production scale of 20,000 kt over a 50-year facility lifetime.

### B.3 Inventory

Tables S4–S7 present the life cycle inventory for producing materials per kilogram of composite production, aligned with the functional unit described in Section B.1. The inventories correspond to the four scenarios analyzed: room-temperature delignified reconstructed wood (Rec-RTD), room-temperature delignified reconstructed wood with a single reuse of the delignification solution (Rec-RTD1), room-temperature delignified reconstructed wood with a two-times reuse of the delignification solution (Rec-RTD2), and room-temperature delignified reconstructed wood with a three-times reuse of the delignification solution (Rec-RTD3).

### B.4 Soluble Lignin

The inventory for calculating the impacts of soluble lignin production is provided in Table S8. In the sensitivity analysis, the assumption of isopropanol recycling was evaluated by also modeling the impacts without recycling, using the inventory inputs listed in Table S9.

### B.5 Modeling Impact of Lignin

The inventory for modeling the environmental impact of lignin was based on Moretti et al.^55^ and is detailed in Table S10. A physical allocation method was applied in this analysis.

### B.6 Modeling Impact of Acetic Acid

The production of acetic acid was modeled using a non-weighted average of the production methods available in the Ecoinvent 3.10 database, including:

- Acrylic acid production (RER)
- Acetic anhydride production, acetaldehyde oxidation (RER)
- Acetic acid production, butane oxidation (RER)
- Acetic acid production, methanol carbonylation (Monsanto), product in a 98% solution state (RER).

### B.7 Environmental Quality Results Rec-RTD3

The environmental quality (EQ) results of Rec-RTD3 are shown in Figure S11 to further highlight and understand the impacts of the relignified material. Results are shown under the aggregated unit potential disappeared fraction of species per year (PDF·year^–1^) for the production of 1 kg of the Rec-RTD3 material.

## Notes

Additional characterization and methodological details including complementary SEM–EDX overlays confirming the localization of brominated impregnating lignin within reconstructed poplar, representative XRD and SAXS patterns, and the evolution of *d*-spacing with hot delignification duration; the three tensile-testing orientations (0°, 45°, and 90°) alongside representative stress–strain curves for Rec-RTD, Rec-2HD, and Rec-6HD; extended descriptions of the material-fabrication parameter ranges, machine learning hyperparameters, and model performance metrics (F1 and *R* ^2^ scores from 5-fold cross-validation); and detailed flowcharts of the End-to-End regression workflow and the overall reconstruction process—together with LCA system boundaries, inventory inputs, and ecosystem quality impacts (PDF)
