# Supplementary material for: Combined Experimental and Machine Learning Study on the Interplay between Delignification and Mechanical Properties for Improved Poplar Wood Reconstruction
Source: ACS Appl Mater Interfaces. 2026 Jan 14;18(3):5882–95. doi: 10.1021/acsami.5c20194 (PMC12862761; doi:10.1021/acsami.5c20194)
Supplement: Supplementary file 1 [file am5c20194_si_001.pdf]

Supporting Information

Combined Experimental and Machine Learning Study on the Interplay between Delignification and Mechanical Properties for Improved Poplar Wood Reconstruction

A. Vahid Movahedi-Rad*[1], Maximilian Ritter[1,2], Alan Colmant[1], Dan Vivas Glaser[1], Sandro Stucki[1,2], Ingo Burgert[1,2], and Guido Panzarasa*[1]

[1]Wood Materials Science, Institute for Building Materials, ETH Zürich, 8093 Zürich, Switzerland.

[2]Laboratory for Cellulose & Wood Materials, Group WoodTec, Empa, Swiss Federal Laboratories for Materials Science and Technology, Überlandstr. 129, 8600 Dübendorf, Switzerland

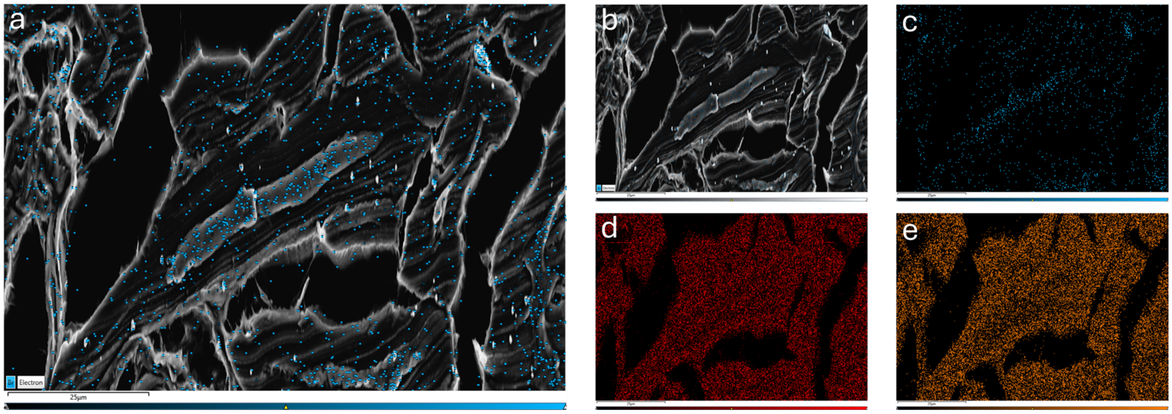

Figure S1: (a) Overlay of SEM micrograph and corresponding EDX map of bromine, showing the localization of impregnating lignin (zones of higher Br concentration) for a representative reconstructed poplar sample. (b) SEM micrograph. EDX maps of (c) bromine, (d) carbon and (e) oxygen. The labeling with bromine was performed according to [1]. The labeling treatment resulted in severe morphological sample damage, such as interface failure and void formation.

*Corresponding author: avahidmovahedirad@ethz.ch
*Corresponding author: guidop@ethz.ch

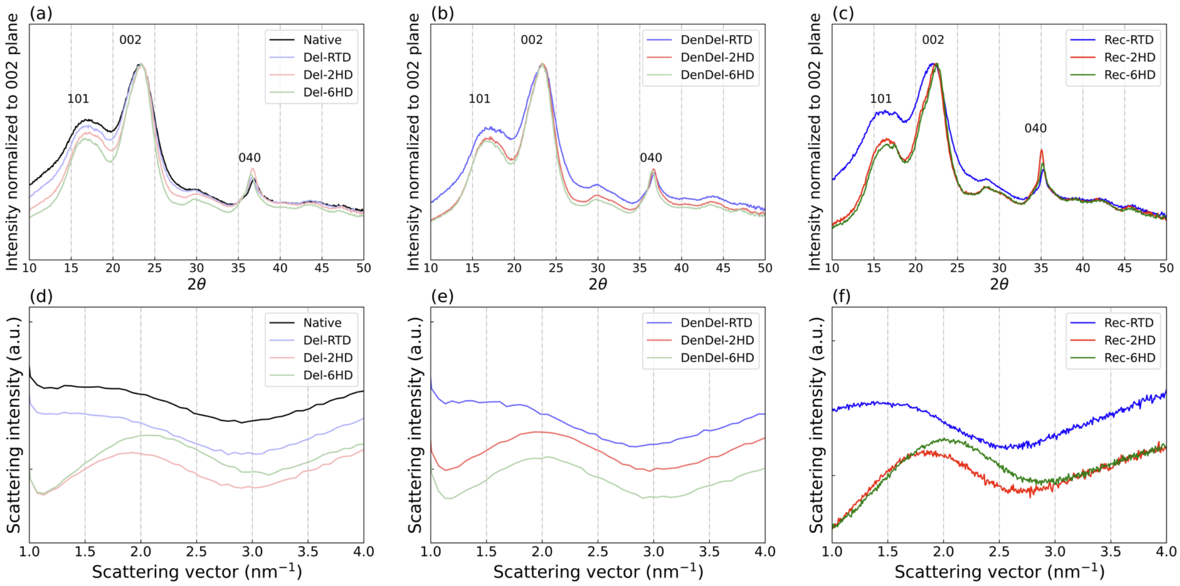

Figure S2: Representative XRD plots of; (a) Native poplar, DelPop-RTD, DelPop-2HD, and DelPop-6HD, (b) DenDelPop-RTD, DenDelPop-2HD, and DenDelPop-6HD (c) Rec-RTD, Rec-2HD, and Rec-6HD. Representative SAXS plots of; (d) Native poplar, DelPop-RTD, DelPop-2HD, and DelPop-6HD, (e) DenDelPop-RTD, DenDelPop-2HD, and DenDelPop-6HD (f) Rec-RTD, Rec-2HD, and Rec-6HD.

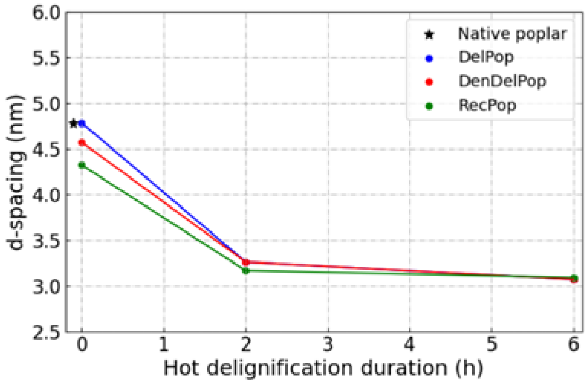

Figure S3: Variation of d-spacing (Eq. 2) versus hot delignification duration in DelPop, DenDelPop, and RecPop.

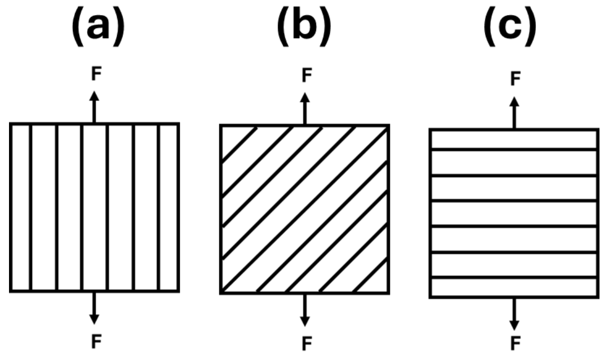

Figure S4: Schematic showing three specimen orientations used for tensile testing at (a) 0° fiber direction, (b) 45° fiber direction, and (c) 90° fiber direction.

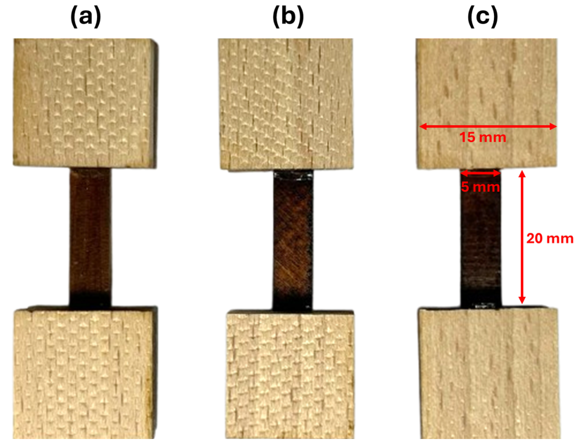

Figure S5: Rec-RTD tensile specimens and their geometries tested at (a) 0° fiber direction, (b) 45° fiber direction, and (c) 90° fiber direction.

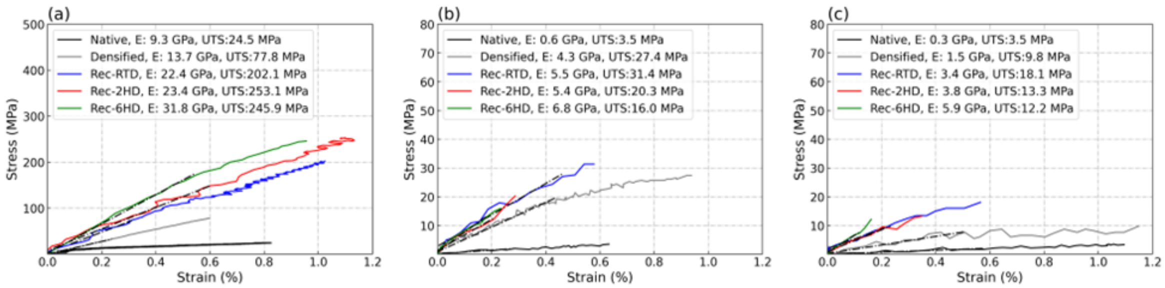

Figure S6: Representative stress–strain curves of Rec-RTD, Rec-2HD, and Rec-6HD at (a) 0°, (b) 45°, and (c) 90° fiber directions.

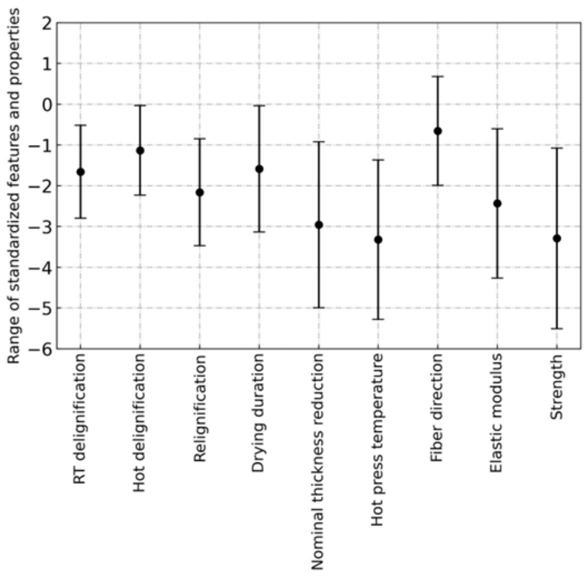

Figure S7: Range of standardized material-fabrication parameters and properties.

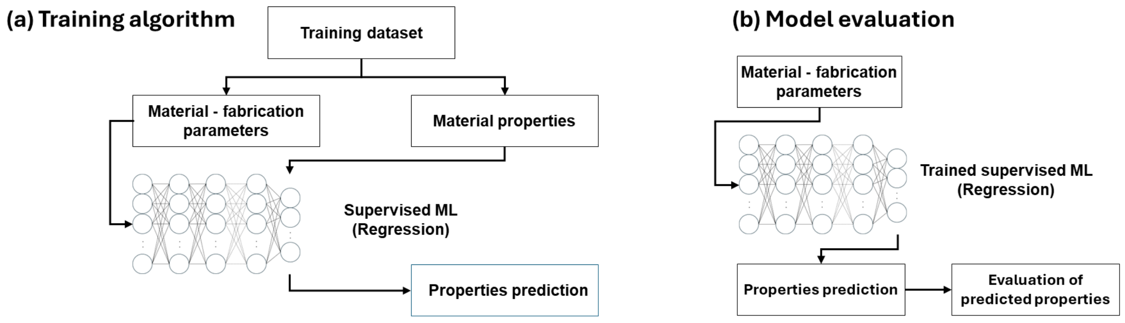

Figure S8: Flowchart for (a) model using an End-to-End supervised regression model, and (b) flowchart for model evaluation.

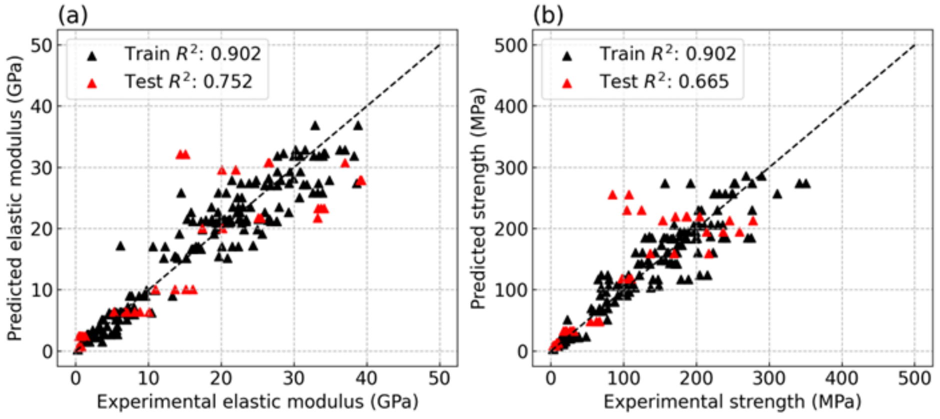

Figure S9: Correlation between predicted and experimental values for (a) elastic modulus, and (b) strength, using End-to-End supervised regression model.

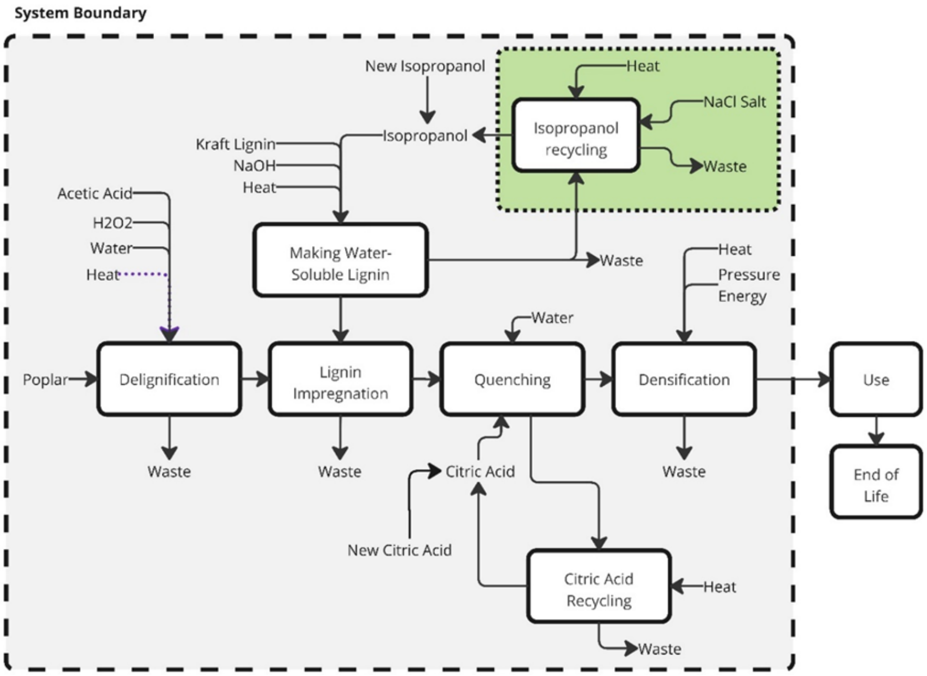

Figure S10: Flow diagram of reconstructed poplar preparation, encompassing all methods described. The outer dotted line indicates the system boundary assessed in LCA. The green section highlights the isopropanol distillation assumption, which is discussed in section 2.2.

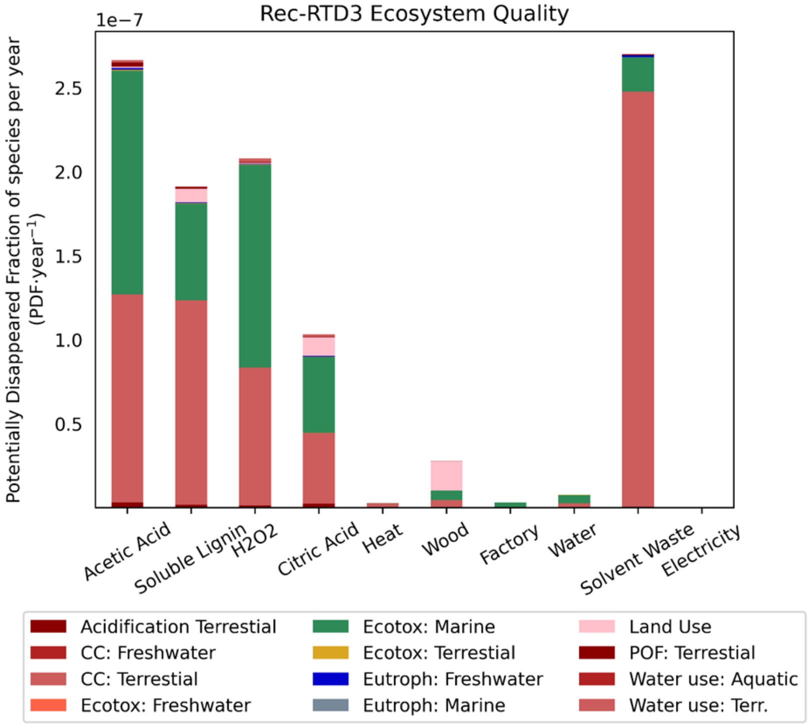

Figure S11: Ecosystem Quality impacts, per material input aggregated following ReCiPe aggregation units of Potentially Disappeared Fraction of species per year (PDF$\cdot$year^{-1}), for the production of 1 kg of Rec-RTD3.

Table S1: Range of material-fabrication parameters explored in this study

Parameters	Variants
RT delignification (h)	0, 6, 24
Hot delignification (h)	0, 1, 2, 4, 6
Relignification (h)	0, 48
Drying duration (day)	0, 1, 2, 3, 5, 6
Nominal thickness reduction (%)	No spacer used, 50, 65, 75, 85, 90, not used
Hot press temperature (°C)	120, 130, 140, 150, not used
Fiber direction (degree)	0, 45, 90

Table S2: Overview of the hyperparameters in classifier FCNN and regressor FCNN

Hyperparameter	Classifier FCNN	Regressor FCNN
Network architecture	64 / 32 / 8	256 / 128 / 64 / 32
Number of cross validation folds	5	5
Activation function	Relu / Relu / Relu / softmax	Relu / Relu / Relu/ Relu / linear
Batch size	64	128
Regularization parameter	0.03	0.0000000001
Learning rate	0.001	0.001
Optimizer	Adam	Adam
Loss function	Sparse Categorical Crossentropy	MSE (Eq. A2)
Patience in Early Stopping	5	100
Class weighting (C1, C2, C3, C4)	0.678, 1.264, 0.866, 1.731	-

Table S3: F1 scores of FCNN classifier and R^2 scores of regressor obtained from 5-fold cross-validation on training and validation sets

	F1 (CV Training)	F1 (CV Validation)	R^2 (CV Training)	$R^2$1 (CV Validation)
Fold 1	1.000	1.000	0.965	0.873
Fold 2	1.000	1.000	0.946	0.959
Fold 3	0.993	0.922	0.929	0.902
Fold 4	0.980	0.974	0.949	0.919
Fold 5	0.919	0.920	0.917	0.942
Mean	0.979	0.963	0.941	0.919
Std	0.034	0.040	0.019	0.034

Table S4: Inventory inputs for modeling the environmental impact of room temperature delignified reconstructed wood (Rec-RTD), normalized per kg of material

Input	Quantity	Availability in the dataset
Wood	2.83 E-03 m^3	Yes, as Market for sawnwood, lath, hardwood, Europe without Switzerland
Soluble Lignin	7.14 E-01 kg	No
Hydrogen Peroxide	8.17 Kg	Yes, as Market for hydrogen peroxide, without water, in 50% solution state
Acetic Acid	12.9 Kg	Yes, but in multiple options, see Table S11
Waste Solvent	26.5 Kg	Yes, as Market for spent solvent mixture, Europe without Switzerland
Water	356 L	Yes, as Market group for tap water, RER
Electricity	9.01 E-03 kWh	Yes, as Market group for electricity, medium voltage, RER
Heat	1.44 MJ	Yes as Heat production, natural gas, at industrial furnace >100 kW Europe without Switzerland
Infrastructure	6.13 E-11 unit*	Yes, as Pulp Factory Construction

*Estimated from the total production of 20000 kt of pulp production estimated by Ecoinvent 3.10 database.

Table S5: Inventory inputs for modeling the environmental impact of room temperature delignified reconstructed wood with one time reuse of delignification solution (Rec-RTD1), normalized per kg of material. Only delignification inventory is shown as all other is equal to Rec-RTD (see Table S4)

Input	Quantity	Availability in the dataset
Hydrogen Peroxide	4.08 Kg	Yes, as Market for hydrogen peroxide, without water, in 50% solution state
Acetic Acid	6.44 Kg	Yes, but in multiple options, see Table S11
Waste Solvent	13.2 Kg	Yes, as Market for spent solvent mixture, Europe without Switzerland

Table S6: Inventory inputs for modeling the environmental impact of room temperature delignified reconstructed wood with two times reuse of delignification solution (Rec-RTD2), normalized per kg of material. Only delignification inventory is shown as all other is equal to Rec-RTD (see Table S4)

Input	Quantity	Availability in the dataset
Hydrogen Peroxide	2.72 Kg	Yes, as Market for hydrogen peroxide, without water, in 50% solution state
Acetic Acid	4.29 Kg	Yes, but in multiple options, see Table S11
Waste Solvent	8.83 Kg	Yes, as Market for spent solvent mixture, Europe without Switzerland

Table S7: Inventory inputs for modeling the environmental impact of room temperature delignified reconstructed wood with three times reuse of delignification solution (Rec-RTD3), normalized per kg of material. Only delignification inventory is shown as all other is equal to Rec-RTD (see Table S4)

Input	Quantity	Availability in the dataset
Hydrogen Peroxide	2.04 Kg	Yes, as Market for hydrogen peroxide, without water, in 50% solution state
Acetic Acid	3.22 Kg	Yes, but in multiple options, see Table S11
Waste Solvent	6.62 Kg	Yes, as Market for spent solvent mixture, Europe without Switzerland

Table S8: Inventory inputs for modeling the environmental impact of 1 kg of soluble lignin assuming distillation

Input	Quantity	Availability in the dataset
Kraft Lignin	1 Kg	No
Water	1.6 Kg	Yes, as Market group for tap water, RER
Isopropanol	0.755 Kg*	Yes, as Market for Isopropanol, RER
Sodium Hydroxide	0.16 Kg	Yes, as Market for Neutralising Agent, Sodium Hydroxide-equivalent, RER
Heat	13.98 Kg	Yes, as Heat production, natural gas, at industrial furnace >100 kW Europe without Switzerland
Solvent Waste	2.36 Kg	Yes, as Market for spent solvent mixture, Europe without Switzerland

*Isopropanol recycling assuming 90% yield.

Table S9: Inventory inputs for modeling the environmental impact of the production of 1 kg of soluble lignin assuming there is no distillation

Input	Quantity	Availability in the dataset
Kraft Lignin	1 Kg	No.
Water	1.6 Kg	Yes, as Market group for tap water, RER.
Isopropanol	7.55 Kg	Yes, as Market for Isopropanol, RER.
Sodium Hydroxide	0.16 Kg	Yes, as Market for Neutralising Agent, Sodium Hydroxide-equivalent, RER.
Solvent Waste	9.15 Kg	Yes, as Market for spent solvent mixture, Europe without Switzerland.

Table S10: Inventory inputs for modeling the environmental impact lignin using the physical allocation by Moretti et al. [2], per kg of lignin produced

Input	Quantity	Availability in the dataset
Sulfuric Acid	0.23 Kg	Yes, as Market for Sulfuric Acid, RER.
Heat	31.5 MJ	Yes, as Heat production, natural gas, at industrial furnace >100 kW Europe without Switzerland.
Liquid Carbon Dioxide	0.3 Kg	Yes, as Carbon dioxide production, liquid, RER.
Sodium Hydroxide	0.107 Kg	Yes, as Market for Neutralising Agent, Sodium Hydroxide-equivalent, RER.
Lime	0.230 Kg	Yes, as Market for Lime, RER.
Lignin Combustion gasses	-1	Modelled from Source Corona et al. [3]
Electricity	10 kWh	Yes, as Market group for electricity, medium voltage, RER.
Water	4850 Kg	Yes, as Market group for tap water, RER.

Table S11: Inventory inputs for modeling the environmental impact of 1 kg of Acetic Acid, as an average of all available in the Ecoinvent 3.11 database

Input	Quantity	Availability in the dataset
Acetic Acid 1	0.25 Kg	Yes, as acrylic acid production, RER.
Acetic Acid 2	0.25 Kg	Yes, as acetic anhydride production, acetaldehyde oxidation, RER.
Acetic Acid 3	0.25 Kg	Yes, as acetic acid production, butane oxidation, RER.
Acetic Acid 4	0.25 Kg	Yes, as acetic acid production, methanol carboxylation (Monsanto), product in 98% solution state, RER.

References

[1] S Saka, P Whiting, K Fukazawa, and DAI Goring. Comparative studies on lignin distribution by uv microscopy and bromination combined with EDXA. *Wood Science and Technology*, 16(4):269–277, 1982.

[2] Christian Moretti, Blanca Corona, Ric Hoefnagels, Marco van Veen, Iris Vural-Gürsel, Tobias Strating, Richard Gosselink, and Martin Junginger. Kraft lignin as a bio-based ingredient for dutch asphalts: An attributional lca. *Science of the Total Environment*, 806:150316, 2022.

[3] Andrea Corona, Mary J Biddy, Derek R Vardon, Morten Birkved, Michael Z Hauschild, and Gregg T Beckham. Life cycle assessment of adipic acid production from lignin. *Green chemistry*, 20(16):3857–3866, 2018.
